# Weather suitability for outdoor tourism in three European regions in first decades of the twenty-first century

**DOI:** 10.1007/s00484-020-01984-z

**Published:** 2020-08-18

**Authors:** Błażejczyk Anna, Pecelj Milica, Skrynyk Oleh, Krzysztof Błażejczyk, Skrynyk Olesya

**Affiliations:** 1 Bioklimatologia. Laboratory of Bioclimatology and Environmental Ergonomy, Łukowska 17/55, 04-133 Warszawa, Poland; 2 Geographical Institute ‘Jovan Cvijić’ Serbian Academy of Science and Arts, Djure Jakšića 9, Belgrade, 11000 Serbia; 3 University of East Sarajevo, Faculty of Philosophy, Alekse Šantića 1, 71420, Pale, Istočno Sarajevo, Republika Srpska Bosnia and Herzegovina; 4 South Ural State University, Institute of Sports, Tourism and Service, Sony Krivoy, 60 Chelyabinsk, Russia; 5 University of Warsaw, Faculty of Geography and Regional Studies, Krakowskie Przedmieście 26/28, 00-927 Warszawa, Poland; 6 Polish Academy of Sciences, Institute of Geography and Spatial Organization, Climate Impacts Laboratory, Twarda 51/55, 00-818 Warszawa, Poland; 7 National University of Life and Environmental Sciences of Ukraine, Heroyiv Oborony, Kyiv, 15 Ukraine; 8 Ukrainian Hydrometeorological Institute, Nauky, Kyiv, 37 Ukraine

**Keywords:** Outdoor tourism, Weather suitability, Poland, Serbia, Ukraine

## Abstract

Outdoor tourism and recreational activities strongly depend on actual meteorological conditions. Traditionally, in three studied regions, the peak of tourists’ streams concentrates in summer months. In the present study, we assess suitability of weather conditions for various forms of outdoor tourism in different regions of Serbia, Poland and Ukraine. Additionally, how the location of the station differentiates temporal patterns of weather suitability will be discussed. To analyse the suitability of weather conditions for various forms of outdoor recreation, we have chosen 23 meteorological stations of the national weather networks which represent different tourism areas and destinations. For each weather station, daily data for the period 2000–2017 of air temperature, relative humidity, total cloud cover and wind speed (at 10 m above ground) for 12 UTC as well as the daily maximum and minimum temperature, precipitation totals and snow cover depth were applied. Suitability of climate for outdoor recreation and tourism is assessed by the Weather Suitability Index (WSI) based on Błażejczyk’s bio-thermal weather classification. The results of research show that passive forms of recreation (sun and air bathing) are preferred mostly in months from May till August or September. For the active forms of recreation, weather in summer months is very oppressive, especially in the resorts located in the south (Serbia, southern Ukraine). Active forms of recreation are preferred there in autumn, winter and spring months.

**Electronic supplementary material:**

The online version of this article (10.1007/s00484-020-01984-z) contains supplementary material, which is available to authorized users.

## Introduction

During the last decades, tourism industry has increased all over the world. According to the United Nations World Tourism Organization (UNWTO), international arrivals have raised from 436 million in 1990 to 1400 million in 2018 (UNWTO, 2019). The most frequently visited places are European countries (710 million in 2018).

Tourism is an important branch of national economies. In Poland, in the years 2006–2016 a number of hotels and other tourism objects has increased to 72%. It is about 2% of the European market. The number of domestic tourists has increased to 57% and international tourists to about 48% (Łysoń 2019). The statistical data for the whole Ukraine cover the period 2000–2013 (2000–2010 based on data from the Ministry of Infrastructure of Ukraine, http://mtu.gov.ua, since 2011—from to the State Statistics Service of Ukraine (2019). From 2014, statistical records do not include the temporarily occupied territories of Crimea, Donetsk, Sevastopol and Luhansk. Thus, in the years 2000–2013 about 55% increase in tourists (61% for foreign and 52% for domestic tourists) was noted, and the last years brought a decrease in tourism activity. In Serbia, the number of tourists has increased in the last 17 years (with decreasing and stagnation phases in the years 2009–2013) and in 2018, 9.3 million overnight stays were registered which is 12.1% more than in 2017. The share of foreign tourists in the total number of overnight stays in 2008 was 19.1%, while in 2018 increased to 39.2% (Statistical Yearbook of the Republic of Serbia 2017).

The time series of tourists’ nights contains very pronounced seasonal fluctuations in three studied regions, with the highest values in the summer season and in January. There are several factors that influence tourism activity. One of the main reasons of increasing tourism is the growing richness of population and transportation availability.

There is seen a great seasonality in tourism over the year. In Poland and in Serbia, the greatest number of tourists is registered in the summer months. In 2018, about 34% hotel services of domestic and foreign tourists concentrate in the months June–August and 53% in the period of May–September (Łysoń 2019). The similar seasonal pattern of tourists’ traffic is also observed in Serbia (Statistical Yearbook of the Republic of Serbia 2017). Such concentration of tourism activity has different reasons (e.g. organisation of school year, holiday habits), and one of them is climate conditions assessed as appropriated for recreation (Andrade et al. 2007, Hamilton 2007, Mansfeld et al. 2007, Scott et al. 2007, Vrtačnik Garbas 2007).

There are hundreds of research and papers considering the influence of various climate elements on the different kinds of tourism. In climate–tourism research, the most frequently used are physiological equivalent temperature (PET, Höppe 1999), perceived temperature (PT, Jendritzky 1990, VDI 2008), apparent temperature (AT, Steadman 1984), heat index (Rothfusz 1990), effective temperature (ET, Baranowska and Gabryl 1981), physiological subjective temperature (PST, Błażejczyk 2007), predicted mean vote (PMV, Fanger 1970), and Universal Thermal Climate Index (UTCI, Błażejczyk et al. 2010). The most frequently used indices were listed and discussed by Epstein and Moran (2006), Błażejczyk et al. (2012) and de Freitas and Grigorieva (2017). While tourism integrally corresponds to the change of the place of stay, special adaptation indices are also used.

Climate information provided to tourists and tourism industry should include not only its general features (mean monthly and annual values of particular climate elements) but also a detailed information regarded to thermal comfort as well as aesthetic and physical weather factors (de Freitas et al. 2008). All of these factors have to be included in an integrated assessment of tourism climate (Matzarakis 2007). These requirements of climate–tourism research represent weather approach which considers the complex of meteorological elements (i.e. the actual weather) influencing the human organism. Several proposals of weather classification have been developed so far (Błażejczyk and Kunert 2011). More recently, there are proposals from Błażejczyk (2007) and Andrade et al. (2007) that are based on human heat balance considerations. There are also the in use DWD (German Weather Service) synoptic classification of weather (Bissoli and Dittmann 2001; Bucher 1991) and Spatial Synoptic Classification (SSC, Kalkstein et al. 1996, Sheridan 2002). However, DWD and SSC classifications are dedicated for general assessment of bioclimate and not for climate–tourism research.

An integral assessment of weather and climate information for tourism is possible by analysis of the Climate-Tourism-Information-Schemes (CTIS) (Matzarakis 2007), Climate Index for Tourism (CIT, de Freitas et al. 2008) and Weather Suitability Index (WSI, Błażejczyk 2007). All base their data on daily meteorological information and human heat balance measures. However, Mieczkowski’s (1985) Tourism Climate Index (TCI) bases on the mean monthly climate data. It is used (after some modifications) for the general assessment of climate usefulness for tourism in continental and global scales (e.g. Amelung and Nicholls 2014; Kovács and Unger 2014).

Temporal and spatial variability is one of the essential features of climate experienced during tourism activity. Tourists’ organisms must adapt to the changing climate stimuli. In general, an increase in differences of climate stimuli intensifies the magnitude of adaptation processes in an organism (Jendritzky and de Dear 2008; Koppe and Jendritzky 2005). There are few attempts of quantitative assessments of bioclimatic contrasts between different sites. The concept of Bioclimatic Distance (BD, Mateeva and Filipov 2003) bases on the comparison of clothing necessary for keeping the heat balance of the human body. The Acclimatization Thermal Strain Index (ATSI) of de Freitas and Grigorieva (2009) is based on heat loss by respiration. Błażejczyk and Vinogradova (2014) proposed the Adaptation Strain Index (ASI) which includes three measures of adaptation processes of an organism to different ambient conditions: water loss, insulation predicted and Universal Thermal Climate Index.

There are many studies reporting bioclimatic potential for recreation and tourism in Poland and in Serbia. General characteristics of whole Poland were done in several publications of Błażejczyk and co-authors (Błażejczyk et al. 2015, 2018, Błażejczyk and Kunert 2010, 2011). There are also researches assessing bioclimatic conditions for tourism in different regions of Poland. As an example of research using WSI approach, we can list papers of Radzka and Dragańska (2015), Miszuk (2008), and Mąkosza (2013). However, Mąkosza and Michalska (2010, 2011) have applied human heat balance–based indices (Subjective Temperature Index, STI and Physiological Strain, PhS) to assess bioclimatic potential for tourism and recreation in western Poland. Recently, several papers have been published in Serbia considering heat budget indices in the context of tourism and recreation. Pecelj et al. (2013) analysed the Weather Suitability Index (WSI), physiological subjective temperature (PST) and physiological strain index (PhS) for different physical activities in tourism context, and Basarin et al. (2018) used physiological equivalent temperature (PET) to study bioclimatic changes in Novi Sad. Bioclimatic conditions of Zlatibor, Belgrade and Loznica were analyzed referring to the Universal Thermal Climate Index (UTCI) by Pecelj et al. (2017) and Pecelj et al. (2018). Stojićević et al. (2016) studied the bioclimate condition of Banja Koviljaca Spa using PET.

To the best of our knowledge, for Ukraine, there are several researches addressed to the influence of climate on tourism activity. For instance, a current state of bioclimatic conditions and their usefulness for tourism in Odesa (the coast of the Black sea) was considered by Katerusha and Matzarakis (2015) by applying the CTIS approach. Seasonal and multiannual changes of PET in Kyiv in the period 1961–2015 was assessed by Shevchenko et al. (2020). General information regarding climate resources can be found in tourism geography and tourism economy publications where authors pay attention to the importance of climate and weather in recreational balneology and spa therapy (Beydyk 2001; Hladkyi and Mirzodaieva 2018).

There are also some attempts to compare bioclimatic conditions in various parts of Europe,—e.g. Nicholls and Amelung (2008) assessed tourism potential using the TCI for Nordic countries and Kovács and Unger (2014) for the central European region. Błażejczyk and Matzarakis (2008) used PST, WSI and CTIS to evaluate climate suitability of Helsinki, Athens, Paris and Cracow. Błażejczyk and Vinogradova (2014) studied the adaptation strain of tourists travelling from central and northern Europe to the Mediteranean. Several papers comparing bioclimatic conditions in various sites in Europe were also published by Błażejczyk and collaborators (Błażejczyk et al. 2010; Błażejczyk and Kunert 2010; Błażejczyk and Błażejczyk 2014; Błażejczyk et al. 2015).

The aim of the present study is to assess suitability of weather conditions for various forms of outdoor tourism and recreation in different regions of Serbia, Poland and Ukraine and to compare how location of the station differentiates temporal patterns of weather suitability.

## Materials and methods

**Fig. 1:**
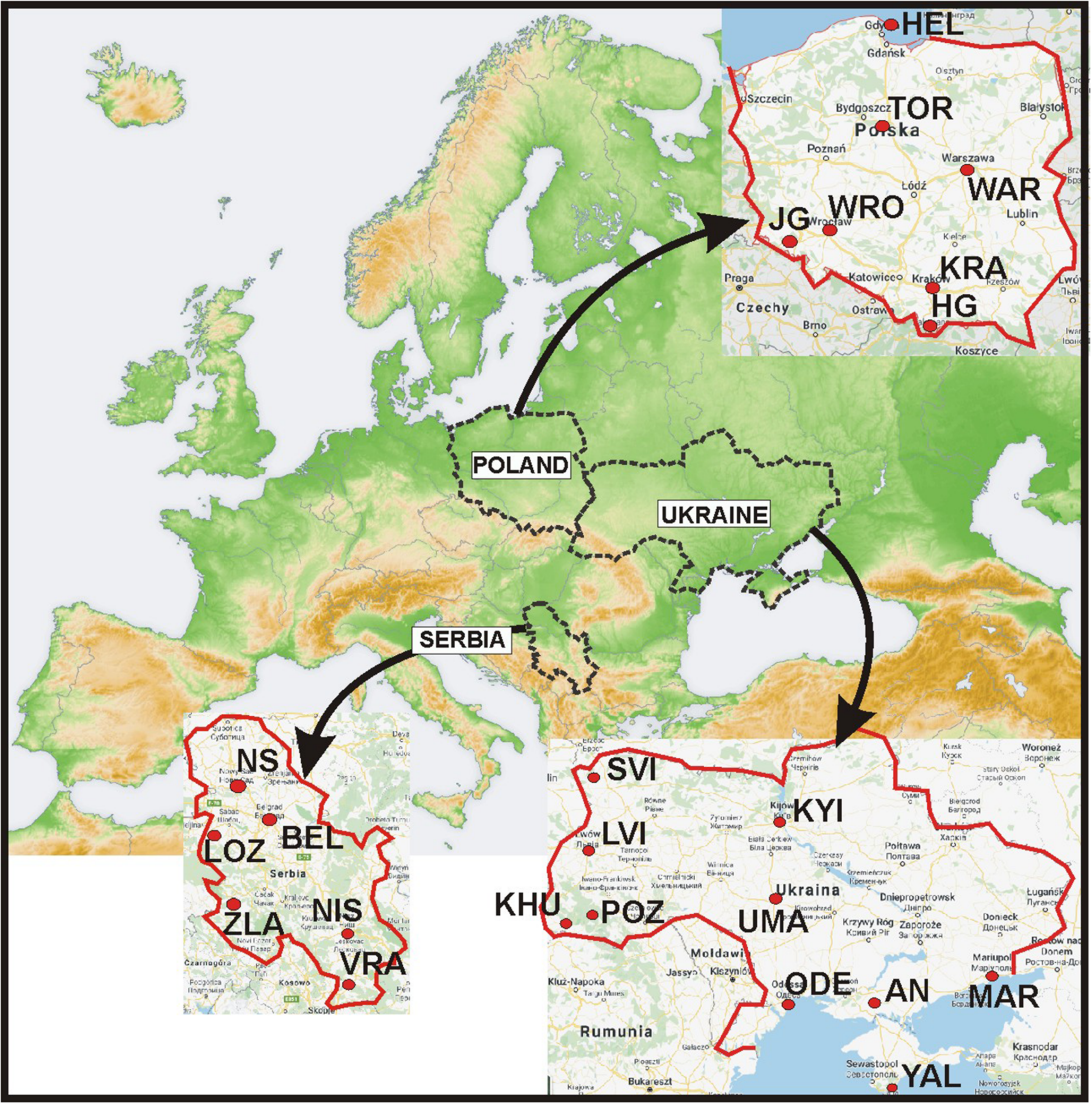
Distribution of studied regions and meteorological stations used in research (abbreviations of stations in Table 1). Source: own elaboration at the map of San Jose—own map, based on the Generic Mapping Tools and ETOPO2, CC BY-SA 3.0, https://commons.wikimedia.org/w/index.php?curid=676986, maps of particular countries at the background of Google maps

To analyse suitability of weather conditions for various forms of outdoor tourism and recreation in Serbia, Poland and Ukraine, we have chosen meteorological stations of the national weather networks: Republic Hydrometeorological Service of Serbia (http://www.hidmet.gov.rs/latin/meteorologija/klimatologija_godisnjaci.php, available June 15, 2020), Institute of Meteorology and Water Management (https://dane.imgw.pl/data/dane_pomiarowo_obserwacyjne/, available June 15, 2020), and Central Geophysical Observatory of the Ukrainian Weather Service (no open access) which represent different tourism areas and destinations (Table 1, Fig. 1). When selecting the weather stations, we have taken into consideration their location due to local landscape which significantly modify regional features of climate (Błażejczyk and Błażejczyk 2014). All the stations are located outside the urban areas and represent regional features of climate. The influence of local factors (as slope exposure, land use, urbanization level, etc.) can be neglected (Table 1).

**Table 1:** Meteorological stations used in the research

Country	Name of station (and abbreviation)	Type of landscape	Köppen-Geiger climate class	Latitude (North)	Longitude (East)	Elevation (m a.s.l.)
Serbia	Novi Sad (NS)	Lowland, river valley	Cfb	45° 20′	19° 51′	85
	Belgrade (BEL)	Lowland, river valley	Cfa	44° 48′	20° 28′	130
	Loznica (LOZ)	Mountain foot	Cfb	44° 33′	19° 14′	125
	Mt. Zlatibor (ZLA)	Mountain	Dfc	43° 44′	19° 43′	1030
	Niš (NIS)	Mountain foot	Cfb	43° 20′	21° 54′	200
	Vranje (VRA)	Mountain valley	Cfb	42° 33′	21° 55′	435
Poland	Hel (HEL)	Sea coast	Dfb	54° 36′	18° 49′	10
	Toruń (TOR)	Lowland, river valley	Dfb	53° 03′	18° 35′	70
	Warszawa (WAR)	Lowland, river valley	Dfb	52° 10′	20° 58′	105
	Wrocław (WRO)	Lowland	Dfb	51° 06′	16° 53′	125
	Kraków (KRA)	Mountain foot	Dfb	50° 05′	50° 47′	240
	Jelenia Góra (JG)	Mountain valley	Dfc	50° 54′	15° 48′	345
	Hala Gąsienicowa (HG)	Mountain	Dfc	49° 14′	20° 00′	1520
Ukraine	Svityaz (SVI)	Lowland, lake coast	Dfb	51° 29′	23° 51′	164
	Kyiv (KYI)	Lowland, river valley	Dfb	50° 23′	30° 32′	166
	Lviv (LVI)	Upland	Dfb	49° 48”	23° 58”	319
	Uman (UMA)	Lowland	Dfb	48° 46′	30° 14′	214
	Khust (KHU)	Mountain foot	Dfb	48° 11”	23° 17”	164
	Pozhyzhevska (POZ)	Mountain	Dfc	48° 09′	24° 32′	1451
	Mariupol (MAR)	Lowland	Dfa	47° 03′	37° 29′	68
	Askaniya Nova (AN)	Lowland	Dfa	46° 27′	33° 53′	28
	Odesa (ODE)	Sea coast	Dfa	46° 27′	30° 46′	42
	Yalta (YAL)	Sea coast	Cfa	44° 29′	34° 09′	66

Source: own elaboration

In general, the stations represent two classes of climate based on the Köppen-Geiger classification (Peel et al. 2007):

- Temperate climates (C—temperature of the hottest month >10 °C and the coldest one from 0 to 18 °C), without dry season (f) and with hot (a), warm (b) or cold (c) summer,

- Cold climates (D—temperature of the hottest month >10 °C and the coldest one ≤0 °C) without dry season (f) and with hot (a), warm (b) or cold (c) summer

For each weather station, daily data of air temperature, relative humidity, total cloud cover and wind speed at 10 m above ground for 12 UTC was applied. We have also used daily data of maximum and minimum air temperature, precipitation totals and snow cover depth. The observational and homogenised data provided by the national weather services of Poland, Serbia and Ukraine cover the period 2000–2017 (the exception is Yalta, where due to temporal occupation of Crimea, data are available only till 2014). For the entire period, data were 100% complete.

Suitability of climate for outdoor tourism and recreation is assessed by the weather suitability index (WSI). It bases on daily meteorological information. WSI is assessed based on bio-thermal classification of weather proposed by Błażejczyk (Błażejczyk 2007, Błażejczyk and Matzarakis 2007, Błażejczyk and Kunert 2011). Weather information is coded by seven digits which represent the following: 1st—thermal sensations, 2nd—radiation stimuli, 3rd—physiological strain, 4th—sultriness, 5th—daily thermal contrast, 6th—rain (snow) fall (>1 mm), 7th—snow cover (>10 cm) (Table 2). Weather characteristics from 1 to 4 refer to bio-thermal conditions during midday hours. Daily thermal contrasts are assessed by the difference between daily maximum and minimum temperatures. In case of precipitation, the best information would be the time of its occurrence during the day. Unfortunately, numerical databases provide only their daily totals and not the time of its occurrence. Thus, rainy/snowy days (i.e. days with precipitation >1 mm) were considered as suitable with limitations for outdoor tourism. The snow cover classification uses criterion of 10-cm depth which is considered as suitable for ski tourism, both in mountain and lowland resorts. For example, the code -2_2C0_011 indicates cold weather (-2) with moderate radiation stimuli (2), cold physiological strain (C), non-sultry (0) with weak daily thermal contrast (0), rain(snow) fall (1) and snow cover (1).

**Table 2:** The scheme of bio-thermal weather classification

	Weather type	Weather subtype			Weather class		
	Thermal sensation	Radiation stimuli	Physiological strain	Sultriness intensity	Daily thermal contrast	Precipitation	Snow cover
	Site of weather indicator						
	1	2	3	4	5	6	7
Weather type	**−***3* (Very cold)						
	**−***2* (Cold)						
	**−***1* (Cool)						
	*0* (Comfortable)						
	*1* (Warm)						
	*2* (Hot)						
	*3* (Very hot)						
Weather subtype		*1* (Weak)					
		*2* (Moderate)					
		*3* (Great)					
			*C* (Cold)				
			*T* (Neutral)				
			*H* (Hot)				
				*0* (Non sultry)			
				*1* (Moderate)			
				*2* (Great)			
Weather class					*0* (Weak)		
					*1* (Significant)		
						*0* (No rain/snow)	
						*1* (Rain/snow >1 mm)	
							*0* (No snow)
							*1* (Snow >10 cm)

Weather indicators set in italics

Source: Błażejczyk (2007)

WSI provides evaluation of each individual weather conditions (with daily resolution) from the point of view of particular forms of recreation:

- sun bathing (WSI_SB),
- air bathing (WSI_AB),
- mild physical activity (e.g. walking, light plays, shopping—WSI_MR),
- intensive physical activity (e.g. football, biking, climbing, jogging, etc.—WSI_AR),
- ski tourism (WSI_ST).

Because of its nature, WSI is not calculated with a mathematical formula but by using a lookup table (see WSI configuration file in BioKlima©2.6 software package, https://www.igipz.pan.pl/bioklima.html). Such a table contains the WSI_xx (where xx = SB, AB, MR, AR, ST) values corresponding to every weather class. Every weather situation is assessed using WSI as follows: 0—unfavourable, 1—favourable (with limitations), 3—very favourable. Table S1 in supplementary materials gives an example of lookup table.

For the particular periods (months, year) individual WSI values for different tourism activities are averaged and categorised as follows (Table 3).

**Table 3:** Range of WSI for different tourism activities

WSI_SB, WSI_AB, WSI_MR, WSI_AR, WSI_ST	Weather suitability for different tourism activities
≤0.50	Unsuitable
0.51–1.20	Moderately suitable
1.21–2.00	Suitable
>2.00	Very suitable

Source: Błażejczyk (2007)

In the present study, we have considered monthly and yearly number of days grouped from categories: moderately suitable, suitable and very suitable. We also verified if mean annual values of this group of categories are statistically significant within the entire country and within different types of landscapes, namely coastal, lowland, lowland river valleys, mountain and mountain food. To discriminate among the means of WSI_SB, WSI_AB, WSI_MR, WSI_AR and WSI_ST, the Fisher’s least significant difference (LSD) procedure at the 95.0% confidence level was applied. The statistical analysis were made with the use of STATGRAPHICS Centurion XVI software package.

The mean monthly values of WSI_SB, WSI_AB, WSI_MR, WSI_AR and WSI_ST were applied to distinguish periods of different suitability (unsuitable, moderately suitable, suitable and very suitable) for various forms of tourism. Such periods are compared between regions and stations.

For the general assessment of weather suitability for tourism, the average value of WSI is applied (Table 4). WSIavg is calculated with the use of following equation:

1 $$ \mathrm{WSIavg}=\mathrm{WSI}\_\mathrm{SB}+\mathrm{WSI}\_\mathrm{AB}+\mathrm{WSI}\_\mathrm{MR}+\mathrm{WSI}\_\mathrm{AR}+\mathrm{WSI}\_\mathrm{ST} $$

**Table 4:** Range of the WSIavg

WSIavg	General weather suitability
≤3.50	Unsuitable
3.51–5.00	Moderately suitable
5.01–6.50	Suitable
6.51–8.00	Very suitable
>8.0	Excellent

Source: Błażejczyk (2007)

## Results

### General features of weather suitability

In Poland, sun bathing can be practised from about 51 days a year in mountain resorts (HG) up to ca. 139 days in coastal locations (HEL) and 140 days in central Poland (WAR). In Ukraine, sun bath weather varies from about 112 days a year at Chornohora mountain ridge (POZ) to 149 days at coastal area of Crimea peninsula (YAL). In Serbia sun baths can be practised from about 77 days a year in Pannonian lowland (NS) up to 111 days in Mt. Zlatibor.

Number of days with weather suitable for air baths varies in Poland from 93 in mountains to about 175–180 in the central lowland (TOR, WAR). In Serbia, such weather is more frequent than in Poland and varies from 146 in Belgrade up to 181 days in Loznica. In Ukraine, occurrence of days of air bath weather is the greatest and change from 164 in the northern part of the country (SVI) to more than 190 days at south-eastern locations (AN, YAL).

Mild recreation activity in Poland can be practised during 274 days in the mountains (HG), 308–312 days in lowland locations (WAR, WRO, TOR) and up to 320 days in coastal resorts (HEL). In Serbia, mild activity days vary from 138 in northern lowland (NS) and up to 188 days a year in mountain resort (ZLA). The most frequent are MR days in Ukrainian tourism areas, and they vary from 270 yearly at the mountain foot (KHU) to 299 at the Black Sea coast (ODE).

Regarding days with weather suitable for active recreation (AR), their greatest number occurs in Poland in the mountains (HG—almost 361 and JG—342 per year). In other resorts, it fluctuates from 324 (KRA) to 337 (HEL). Less frequent AR days are noted in Ukraine and vary from 260 at the Crimea coast (YAL) to 337 at the mountain station (POZ). The smallest number of AR days occur in Serbian resorts, from 112 yearly in Niš to 159 in Mt. Zlatibor.

Ski tourism can be practised mostly in elevated mountain locations. For example, ST suitable weather occurs at the HG station 154 days, at POZ, 115 days and at ZLA, 33 days a year. In mountain valleys (JG), such weather occurs about 31 days a year. Similar number of ST days is observed at eastern locations in Ukraine (KYI, KHU, UMA, LVI). However, at southern locations, both in Serbia and in Ukraine, there are less than 8 days a year with ST weather (Table 5).

**Table 5:** Annual number of days with weather suitable for different forms of outdoor tourism (2000–2017)

Station	Outdoor tourism forms				
	SB	AB	MR	AR	ST
Poland					
HEL	138.6	175.8	319.8	337.4	17.6
TOR	129.5	175.1	311.8	329.4	18.1
WAR	139.7	179.9	307.7	326.9	23.5
WRO	111.5	162.4	308.8	330.6	13.4
KRA	128.4	168.1	313	324.1	19.8
JG	97.8	152.6	308.9	342.1	31.3
HG	50.8	93.4	274.2	360.6	154.2
Serbia					
NS	77.1	164.3	138.4	119.1	2.9
BEL	86.9	146.6	141.9	120.8	4.7
LOZ	109.7	181.2	165.9	116.6	7.6
NIS	95.9	171.9	145.7	112.2	6.1
VRA	85.1	152.9	148.7	128.6	3.9
ZLA	110.9	173.0	188.2	158.7	32.6
Ukraine					
SVI	120.1	164.4	294.1	306.6	24.3
KYI	130.7	179.2	291.2	310.4	36.9
LVI	141.7	188.4	296.1	323.4	29.0
POZ	111.8	168.1	294.8	337.9	115.2
KHU	130.3	180.4	269.2	273.2	30.1
UMA	117.5	169.4	285.9	296.0	38.1
AN	145.8	200.4	295.6	299.4	1.8
MAR	130.3	178.9	286.7	292.9	6.7
ODE	135.8	185.4	298.8	294.9	5.0
YAL	149.3	193.9	283.3	260.4	1.5

For abbreviations of stations, see Table 1

Source: own elaboration

There were analyses on how weather conditions for tourism differ inside studied areas. In this purpose, differences between annual numbers of days suitable for particular forms of outdoor tourism were compared. The most unified weather conditions occur in Serbia which is the smallest, compact country. Frequency of days suitable for various forms of tourism differ significantly only between 20 and 60% of stations. Only the weather in Zlatibor Mt. is significantly different than in other stations. The greatest spatial variation (for 60–100% of stations) is observed in the case of MR and AR weather frequency. The most differentiated weather occurs in Ukraine, the biggest country with great longitudinal and latitudinal extent. This is the cause that in every station, weather differs significantly in comparison to 60–100% of the remaining locations. The greatest weather individuality, in comparison to 82% of other stations, is observed in the mountain area of Eastern Carpathians. The biggest spatial conformity (only 53% differences with remaining stations) occurs in the case of MR weather. In Poland, the most unique weather is observed in mountain areas (HG, significantly different than in all other stations). Comparing types of tourism, the greatest individuality was found for sun bathing weather (Table 6). For a detailed information of inter-station differences in the number of days suitable for SB, AB, MR, AR and ST, see Tables S2, S3 and S4 in supplementary materials.

**Table 6:** Percentage of stations, in particular countries, with statistically significant differences of the annual amount of days suitable for various forms of outdoor tourism

Station	SB	AB	MR	AR	ST	Mean
Poland						
HEL	83.3	50.0	50.0	83.3	16.7	56.7
TOR	83.3	33.3	50.0	66.7	16.7	50.0
WAR	83.3	50.0	50.0	66.7	16.7	53.3
WRO	100.0	66.7	50.0	66.7	33.3	63.3
KRA	83.3	33.3	100.0	100.0	16.7	66.7
JG	100.0	100.0	50.0	83.3	33.3	73.3
HG	100.0	100.0	100.0	100.0	100.0	100.0
Mean	90.5	61.9	64.3	81.0	33.3	x
Serbia						
NS	40.0	60.0	80.0	60.0	20.0	52.0
BEL	40.0	60.0	100.0	60.0	20.0	56.0
LOZ	40.0	60.0	80.0	100.0	20.0	60.0
NIS	40.0	60.0	80.0	100.0	20.0	60.0
VRA	80.0	60.0	60.0	60.0	20.0	56.0
ZLA	80.0	60.0	100.0	100.0	100.0	88.0
Mean	53.3	60.0	83.3	80.0	33.3	x
Ukraine						
SVI	88.9	77.8	33.3	77.8	66.7	68.9
LVI	66.7	66.7	44.4	88.9	44.4	62.2
KY1	77.8	77.8	22.2	88.9	55.6	64.4
POZ	88.9	77.8	44.4	100.0	100.0	82.2
KHU	77.8	77.8	100.0	100.0	55.6	82.2
UMA	77.8	77.8	66.7	66.7	66.7	71.1
AN	66.7	88.9	44.4	55.6	66.7	64.4
ODE	77.8	77.8	55.6	66.7	66.7	68.9
YAL	77.8	66.7	55.6	100.0	66.7	73.3
MAR	77.8	77.8	66.7	66.7	66.7	71.1
Mean	77.8	76.7	53.3	81.1	65.6	x

For abbreviations of stations see Table 1

Source: own elaboration

### Seasonal cycle of weather suitability

Seasonal patterns of weather differ between regions and between locations. In general, the greatest seasonality is well seen for sun (SB) and air (AB) bathing. In winter months (DJF) in majority of the locations, there are not days suitable for sun and air baths. They sporadically appear only at stations situated in Cfa and Cfb climate zones (YAL, NS, BEL, LOZ, NIS). However, in winter months, very frequent are days with weather suitable for mild (MR) and active (AR) recreation (20–30 days per month, depending on station). In the summer season (JJA), frequency of sun and air bathing days increases. In majority of the stations in the summer months, one can observe a great decrease in days suitable for MR and AR activities. It is caused by increased air temperatures during particular summer days. Ski tourism (ST) can be practiced mostly in mountain tourism resorts from December till March or April.

**Fig. 2:**
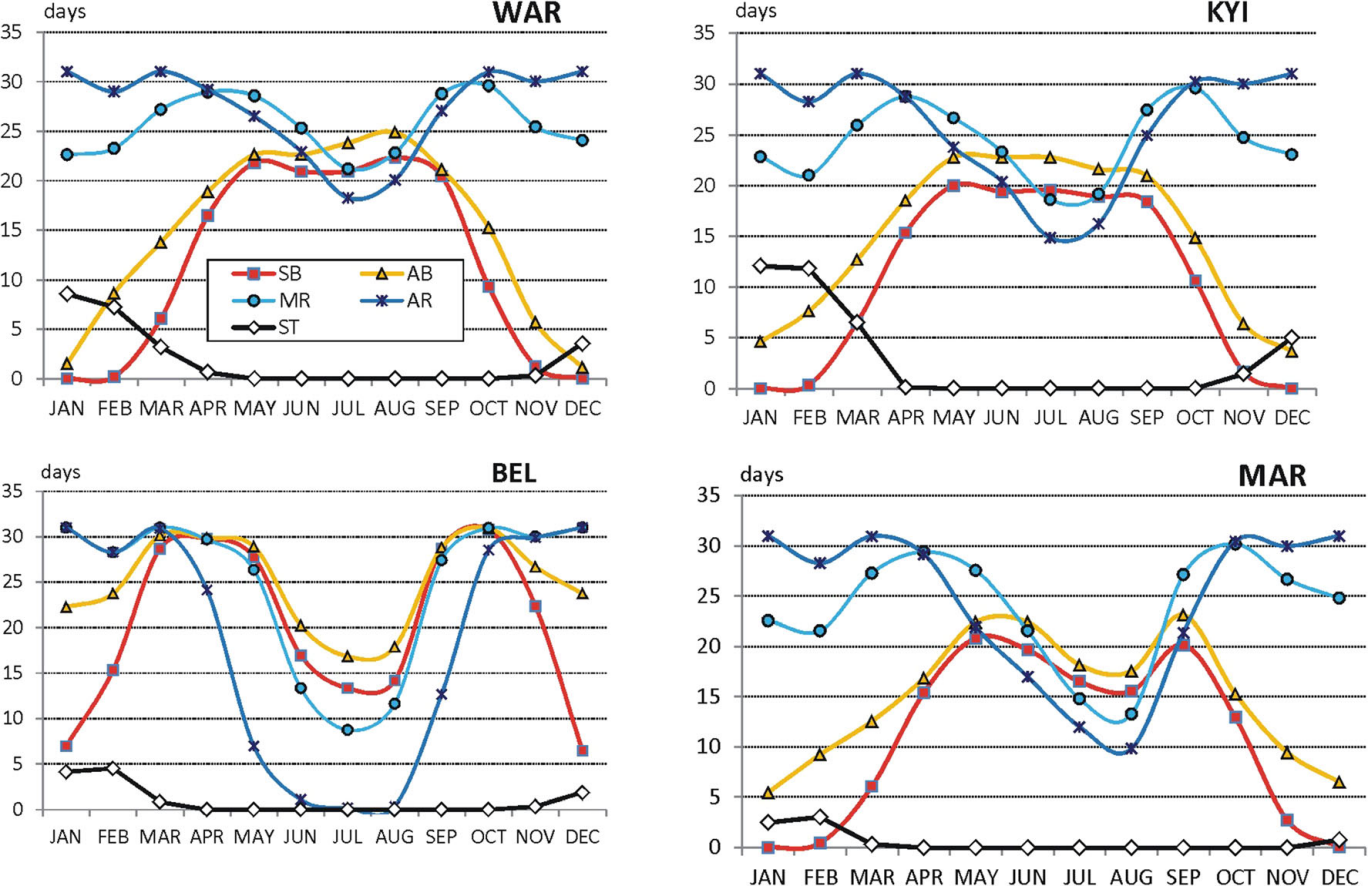
Frequency (days per month) of weather conditions suitable for different forms of outdoor tourism: sun baths (SB), air baths (AB), mild physical activity (MR), intensive physical activity (AR), ski tourism (ST), in selected lowland river valleys stations, 2000–2017 (for abbreviations of stations, see Table 1) Source: own elaboration

The above remarks indicate only main seasonal features of weather in temperate and cold climate zones. Most of them is typical for stations located at lowland landscape (both, at river valleys and flat areas). The seasonal changes of SB and AB weather is the best seen in most northern locations (e.g. WAR, Poland). In most southern locations (e.g. BEL, Serbia), a very strong reduction of days with weather suitable for all forms of recreation is pronounced in summer. At lowland Ukrainian stations, seasonal patterns of weather differ depending on longitudinal location. While in Kyiv (KYI, central part of the country), seasonal weather patterns are similar to those observed in Poland then in the eastern location (MAR), the winter to summer differences in SB, AB, MR and AR are the greatest. It is probably caused by exposition of that region to severe cold arctic air mass in winter and to severe hot tropical air mass in summer months (Fig. 2).

**Fig. 3:**
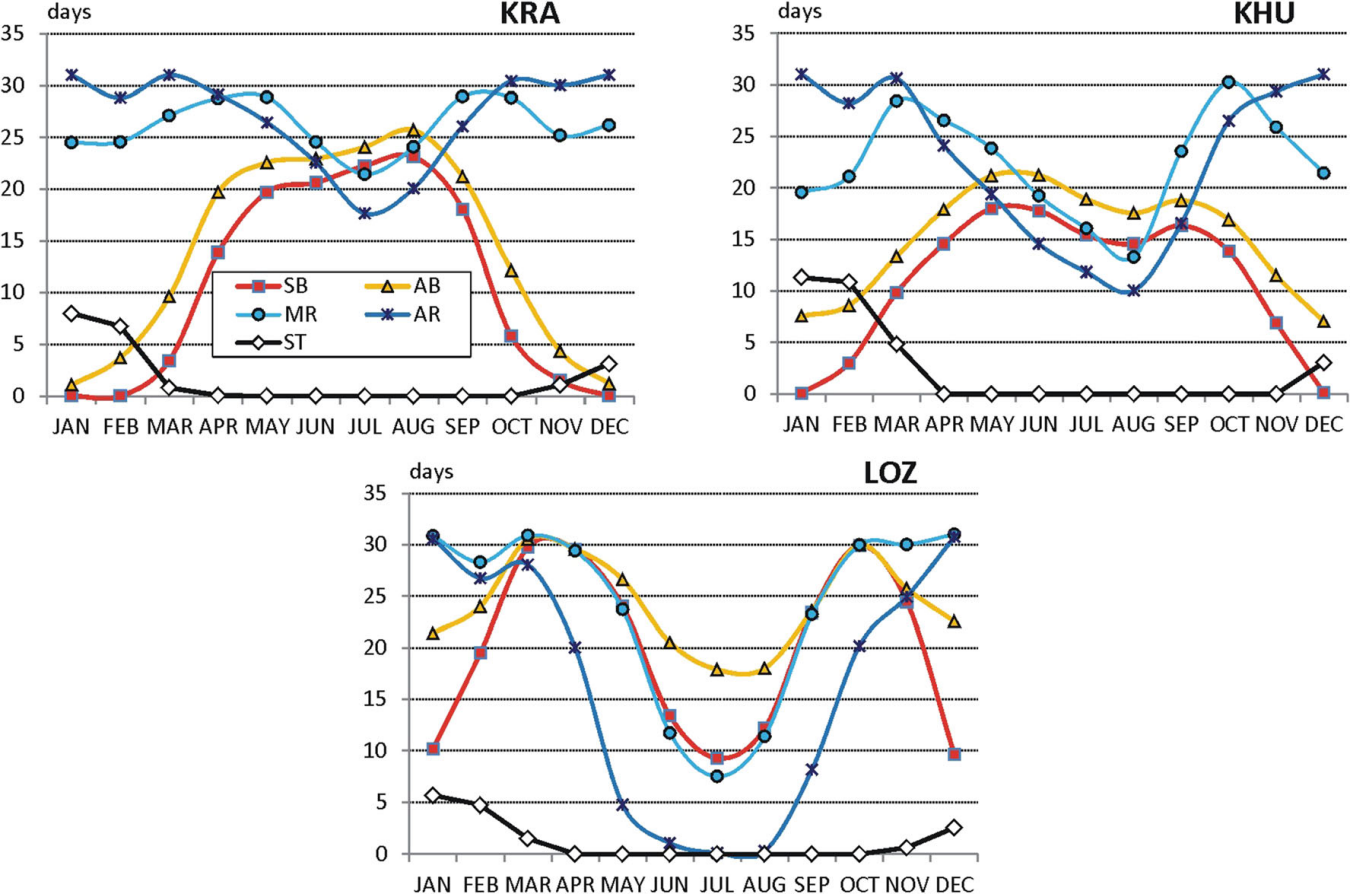
Frequency (days per month) of weather conditions suitable for different forms of outdoor tourism in selected mountain foot stations, 2000–2017, (explanations on Fig. 2, for abbreviations of stations, see Table 1) Source: own elaboration

Increased reduction of days with MR and AR weather is also observed at resorts located in mountain foot areas in Ukraine and Serbia (KHU, LOZ). Moreover, in most southern resorts (LOZ), a great decrease of SB and AB days is pronounced as well (Fig. 3).

**Fig. 4:**
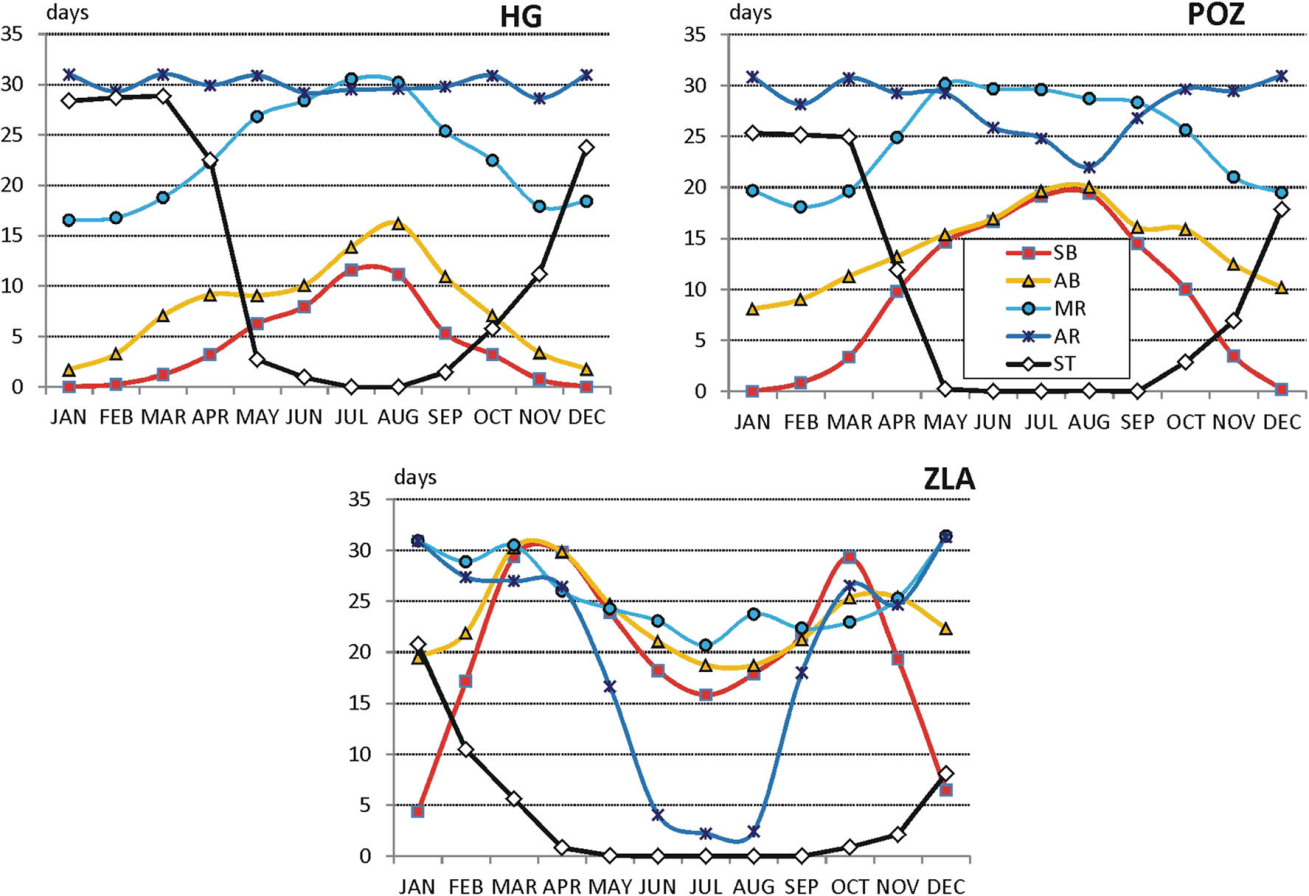
Frequency (days per month) of weather conditions suitable for different forms of outdoor tourism in selected mountain stations, 2000–2017 (explanations on Fig. 2, for abbreviations of stations see Table 1) Source: own elaboration

Regarding mountain stations, there are similarities between Polish Tatras (HG) and Ukrainian Chornohora (POZ). In the summer season, there is an observed culmination of days with SB (12–20 days monthly), AB (17–20 days) and MR (28–31 days monthly) weather. A slight reduction of AR days (to about 22 in August) is observed only in Ukrainian Carpathians (POZ). A contrasting seasonal pattern of weather suitability occurs in Serbian mountains (ZLA). SB and AB weather are most frequent in spring (March–April) and in autumn (September–October), up to 30 days monthly. In summer, such weather is considerably reduced to 15–20 days monthly. At the same time, active recreation can be practised only during 3–5 days a month. The best conditions for ski tourism occur in Tatry Mts. (HG). In the period from January to March, ST activity is possible at 28–29 days monthly. In Chornohora (POZ), there is about 25 ST suitable days. Good ST conditions occur also in December. In Serbia (ZLA), one can plan an ST activity only during 20 days in January, 10 days in February, 8 days in December and 5 days in March (Fig. 4).

**Fig. 5:**
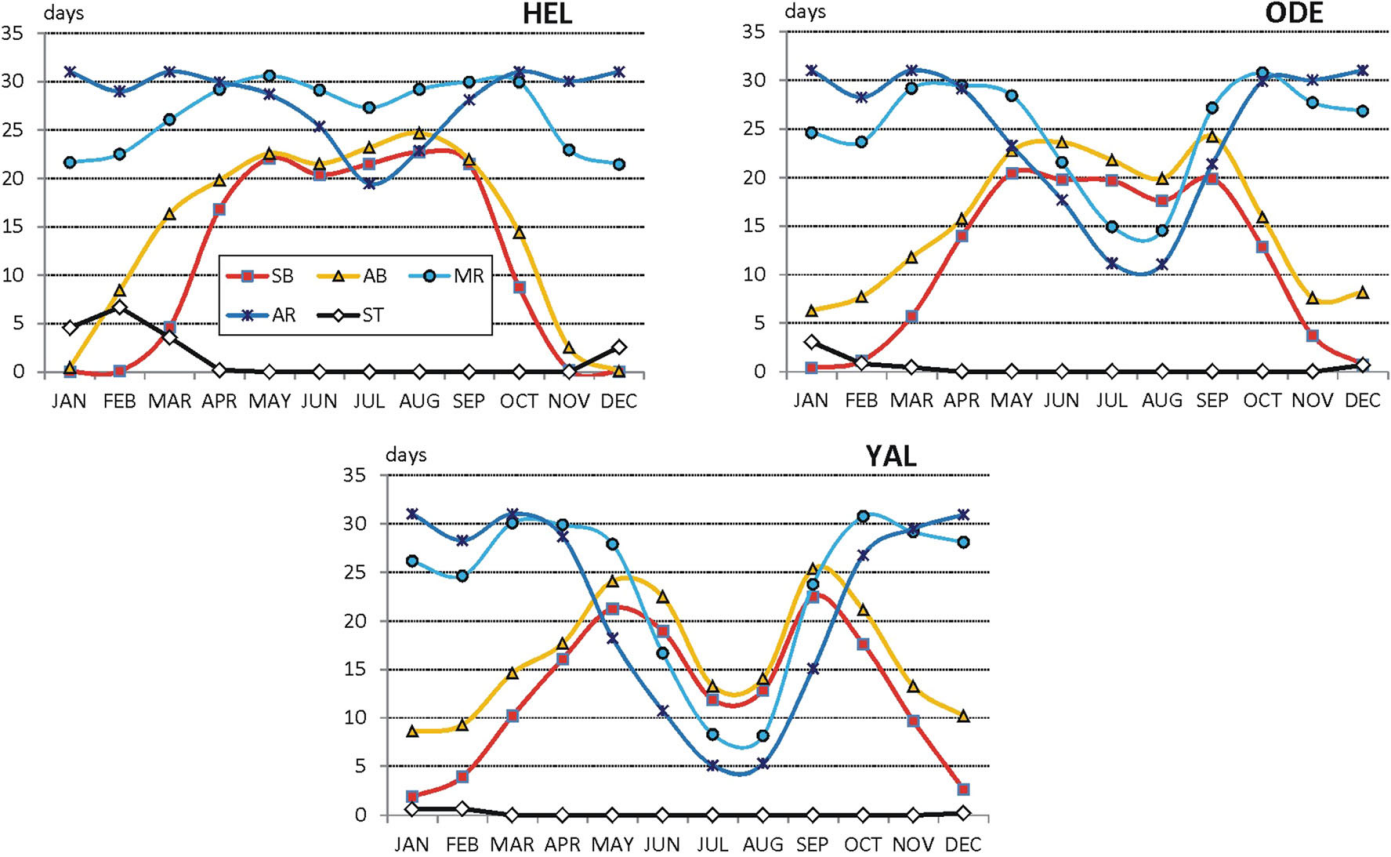
Frequency (days per month) of weather conditions suitable for different forms of outdoor tourism in selected coastal stations, 2000–2017, (explanations on Fig. 2, for abbreviations of stations, see Table 1) Source: own elaboration

Seasonal patterns of weather at coastal resorts can be analysed only for Poland and Ukraine, while Serbia has no access to sea coast. When comparing Polish (Hel) and Ukrainian (Odesa, Yalta) stations, one can see great differences in the annual run of weather suitable for various forms of recreation. At the Baltic coast (HEL), summer decline is not observed in mild recreation weather types (MR), and reduction of the AR weather is relatively small. There is also experienced summer peak of the frequency of SB and AB weather. At the Black Sea coast, summer decline of AR and MR weather is great (due to high temperatures), and in Yalta, active forms of recreation can be practised only 5–10 days a month. Slightly less severe summer weather is observed in Odesa. Typical for this region is also summer reduction of weather suitable for sun and air bathing. In ODE, SB and AB can be applied for about 20 days a month and in YAL, only 12–15 days monthly (Fig. 5).

### Types of landscape and weather suitability

The analysis of annual cycles of the frequency of days suitable for various forms of tourism suggest significant differences between stations located in similar landscape types but in different areas. Thus, statistical significance between annual frequency of SB, AB, MR, AR and ST weathers was verified. At coastal stations, weather conditions differ significantly between Polish (HEL) and Ukrainian (ODE, YAL) resorts for almost all forms of tourism. The same is the concern in lowland, mountain foot and mountain summit stations in studied countries (Table 7). The exception is weather suitable for ski tourism which often does not differ between mountain foot stations in compared areas. An opposite situation was found in the case of stations located in lowland river valleys. Very often, the annual number of SB, AB, MR, AR and ST days do not differ significantly. There are surprisingly similar weathers at stations located far away from each other (e.g. TOR and MAR or TOR and BEL). This needs more detailed research in the future. Mostly differentiated are weather conditions suitable for active forms of tourism (AR) which do not differ only between TOR and WAR but also between WAR and WRO (Table 8).

**Table 7:**
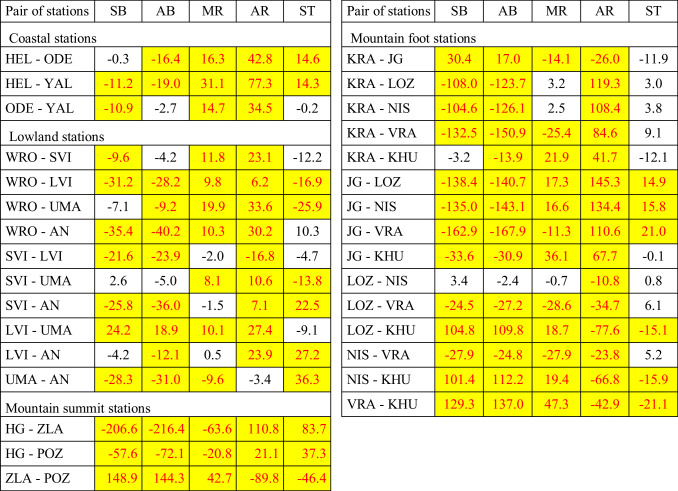
Mean differences in annual frequency of days suitable for particular tourism activities

Differences statistically significant are marked in yellow

(SB sun bathing, AB – air bathing, MR – mild physical activity, AR – intensive physical activity, ST – ski tourism) between coastal, lowland, mountain foot and mountain summit stations, 2000–2017

(for abbreviations of stations see Table 1)

Source: own elaboration

**Table 8:**
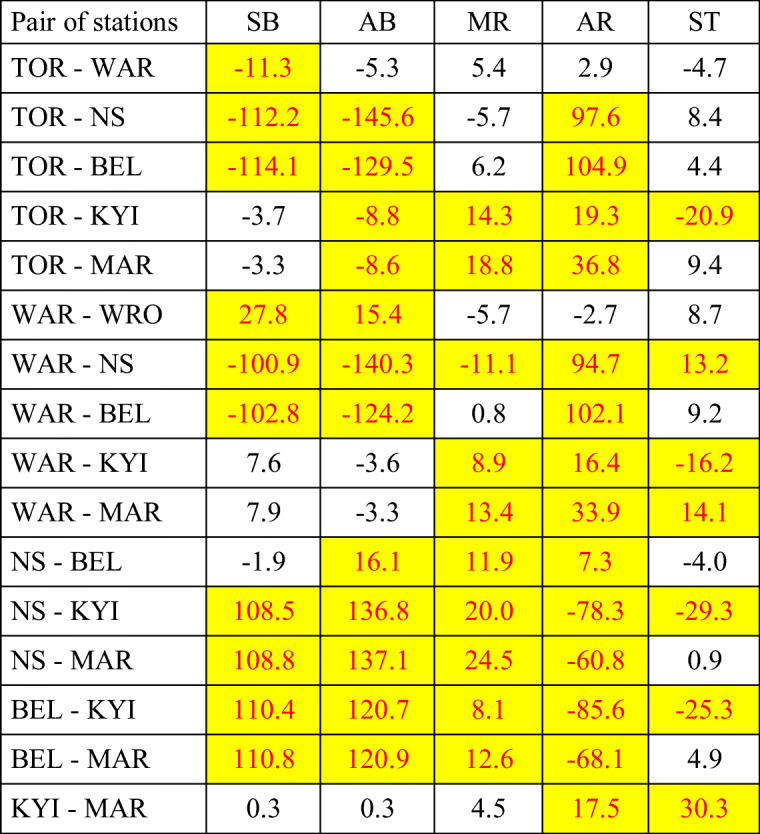
Mean differences in annual frequency of days suitable for particular tourism activities

Differences statistically significant are marked in yellow

(SB – sun bathing, AB – air bathing, MR – mild physical activity, AR – intensive physical activity, ST – ski tourism) between stations located in lowland river valleys, 2000–2017 (for abbreviations of stations see Table 1)

Source: own elaboration

### Periods of weather suitability

#### Sun bathing

The longest period of sun bathing was found for Serbian stations. In majority of tourism resorts, it lasts 12 months a year with different suitability. In all stations, very suitable weather occurs in spring (March–April). In autumn, sun bathing period covers September and October (in Niš, October and November). Sun bathing is unavailable only in three resorts: NS and ZLA in December–January (because of the increased number of cold days in Mt Zlatibor and frequent winds in the Pannonian region during the winter) and in NIS in July (due to increased number of very hot days).

**Fig. 6:**
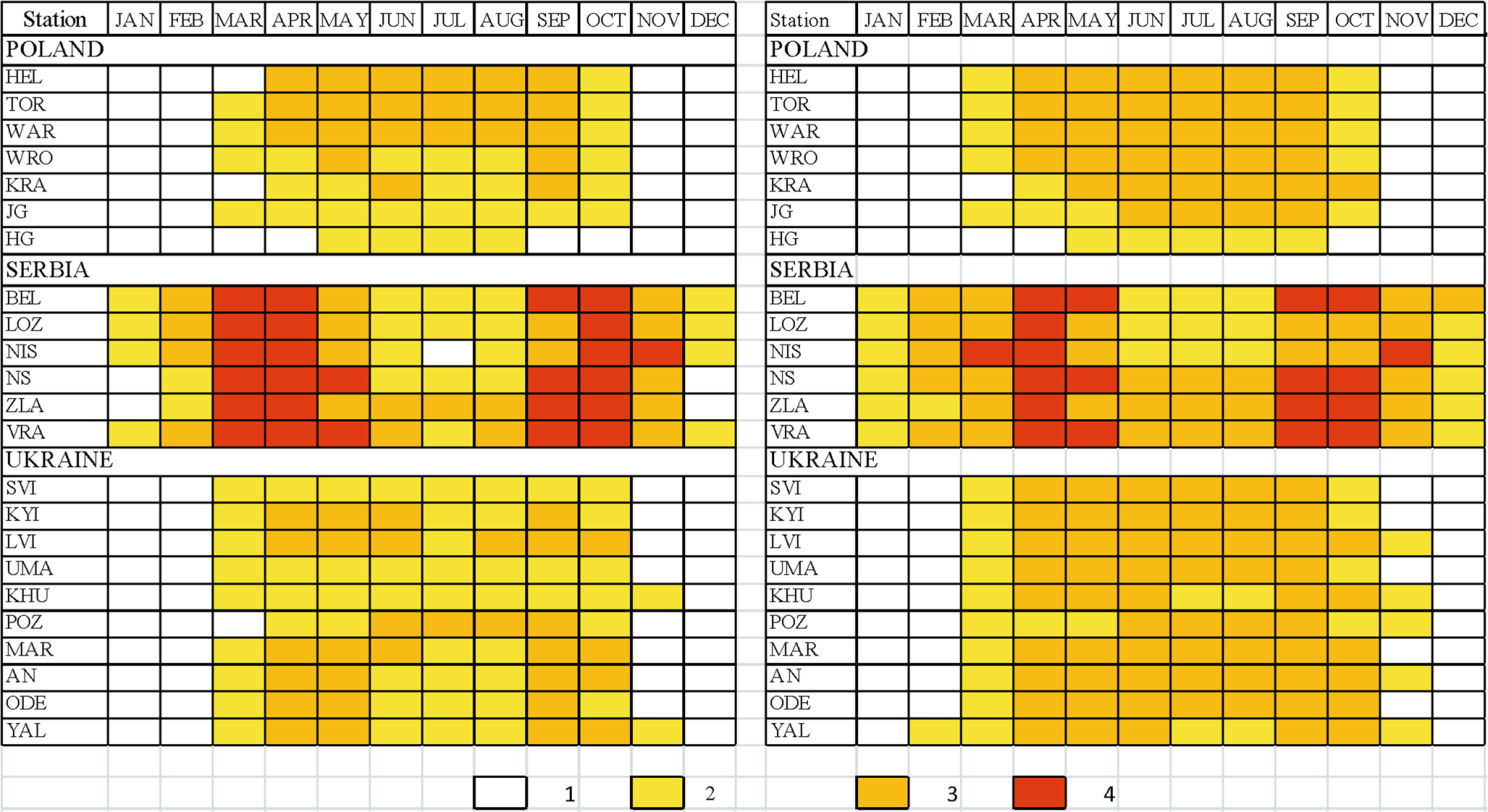
Periods of different weather suitability for sun bathing (left panel) and air bathing (right panel); 1—not suitable, 2—moderately suitable, 3—suitable, 4—very suitable (for abbreviations of stations see, Table 1) Source: own elaboration

In Poland and in Ukraine, sun bathing period is considerably shorter than in Serbia and lasts 7–8 months (mostly from March till October), except elevated parts of mountains (HG) where moderately suitable period covers only 4 months (May–August). It is necessary to add that in the southern part of Poland dominates the moderately suitable sun bathing weather. In central and northern Poland, weather suitable for sun baths covers the period from April till September. Such suitable weather in Ukrainian tourism resorts is mostly observed in spring (April–May) and in autumn (September–October) (Fig. 6).

#### Air bathing

The longest AB period occurs in Serbia, and it lasts the whole year round. However, the best months for air bath are April and May as well as September and October. In winter (December–January), the conditions are only moderately suitable due to relatively great frequency of rainy and cool weather. Again, a very similar seasonal pattern of SB weather was found for Poland and Ukraine. In general, air bathing can be practised there from March till October. The shortest (from May till September) is AB period at elevated mountain resorts (HG) and the longest one at the Crimea coast (YAL), from February till November (Fig. 6).

#### Mild recreation

In Poland, mild recreation can be practised the whole year round, and only in winter (December–January), WSI_MR values indicate moderately suitable conditions (in high mountains, such period lasts from October till March). In the northern part of the region, the period of very suitable conditions occurs in April and September. Similar to Poland, seasonal pattern of mild recreation period is observed in Ukraine. Unsuitable conditions occur only in very southern-located Yalta region in July and August due to very high air temperature.

**Fig. 7:**
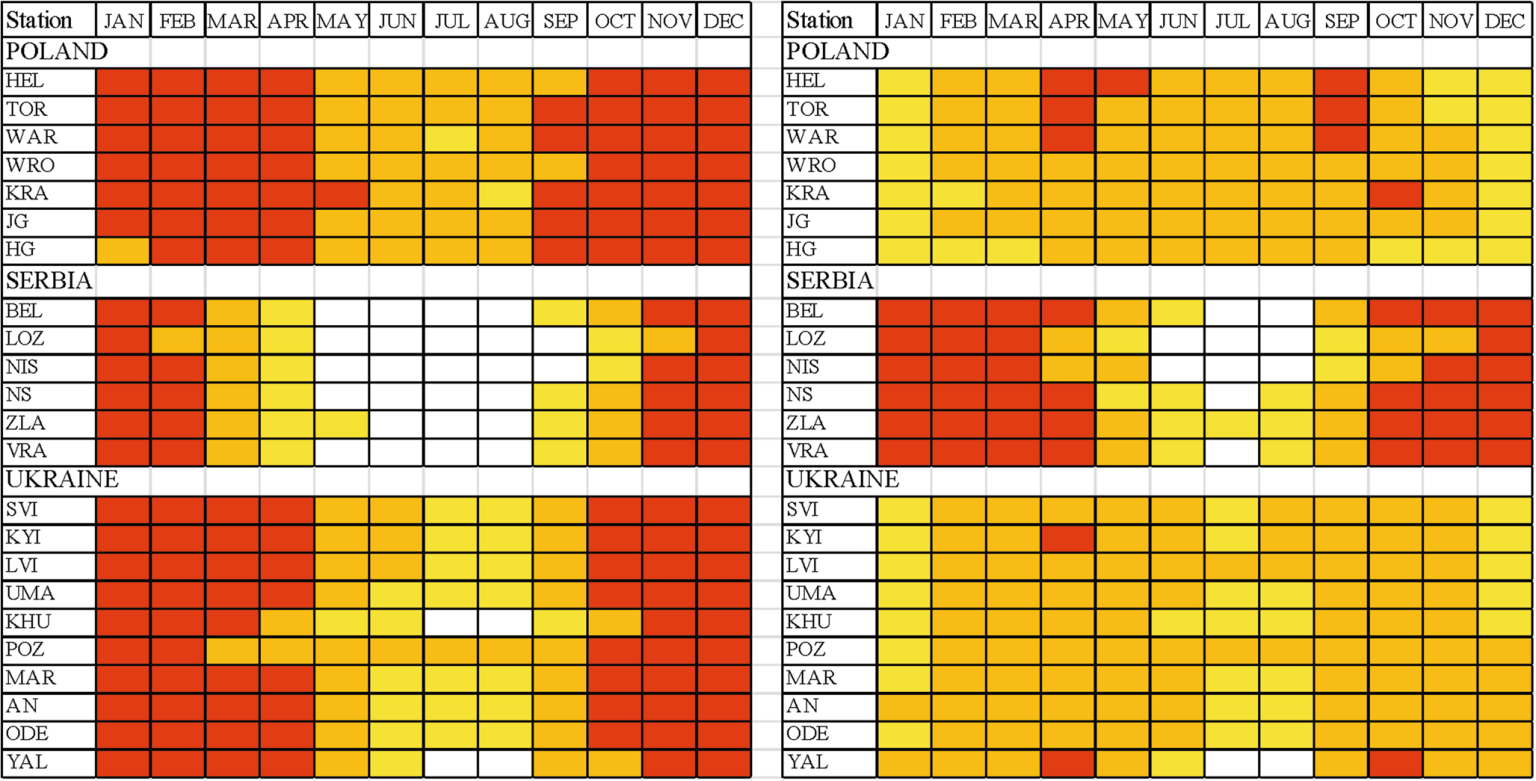
Periods of different weather suitability for mild recreation (left panel) and active recreation (right panel); explanations on Fig. 6. Source: own elaboration

In summer (mostly in July and in some locations in JJA), weather unsuitable for mild recreation is observed in Serbian tourism resorts, except mountain station at Mt. Zlatibor. However, the period of very suitable MR weather in Serbia lasts generally from October till April and is considerably longer then in Poland and Ukraine (Fig. 7).

#### Active recreation

The best conditions to practice active forms of recreation occur in the Polish tourism resorts. In the period from May till August, weather is suitable for active recreation. However, very suitable weather lasts generally from September till April.

Slightly worse AR conditions occur in the Ukrainian resorts. Very suitable weather lasts from October till April (1 month shorter than in Poland). In the summer months (JJA), the conditions are only moderately suitable for active recreation (the exception is Chornohora mountains, POZ). In two stations (KHU and YAL), July and August are unsuitable for AR.

The shortest AR period was found for the Serbian tourism resorts. Very suitable conditions occur only from November till February and unsuitable weather predominates from May till August or even September (Fig. 7).

#### Ski tourism period

**Fig. 8:**
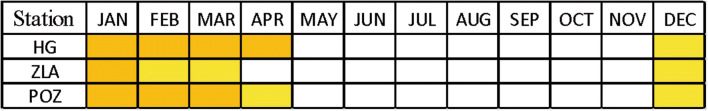
Periods of different weather suitability for ski tourism; explanations on Fig. 6. Source: own elaboration

Ski tourism period can be considered only in the case of mountain resorts. In Poland (HG) and in Ukraine (POZ), ST period lasts from December till April. In Serbia (ZLA), weather conditions suitable or moderately suitable for ski tourism are 1 month shorter (December–March, Fig. 8).

#### General assessment of weather suitability

**Fig. 9:**
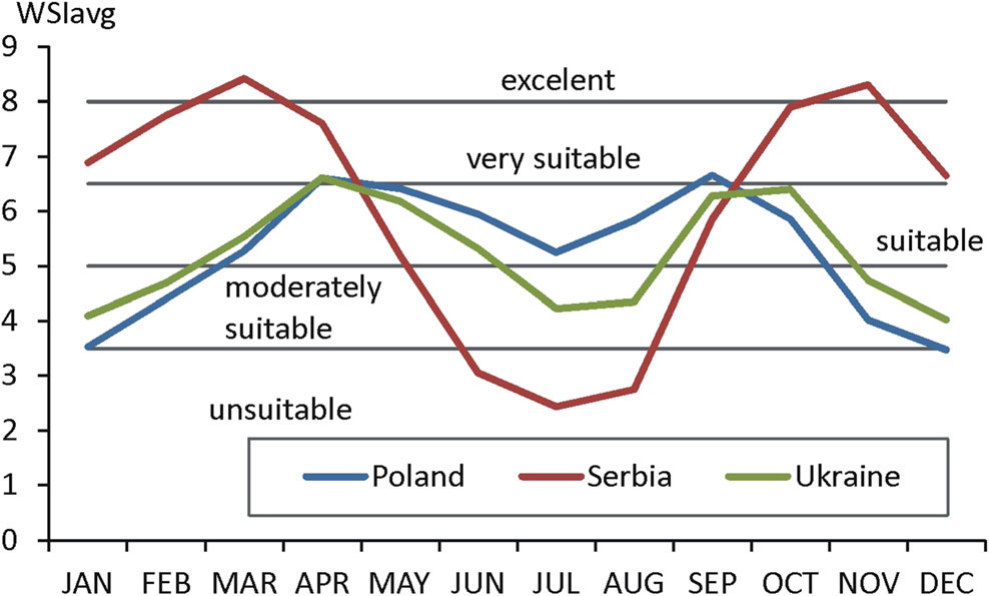
Yearly course of spatially averaged values of WSIavg in studied countries, 2000–2017. Source: own elaboration

The results presented in the previous section show great seasonal differentiation of weather in 3 studied areas: Poland, Serbia and Ukraine. The differences are seen in the case of every form of tourism. To compare how in general weather differs between 3 studied areas, the spatially averaged WSIavg values were calculated for particular countries. In every country, there are observed two maxima (spring and autumn) and two minima (winter and summer) of weather suitability (Fig. 9). The greatest annual variability occurs in Serbian tourism resorts. From October till April, with peaks of excellent weather in March and November, conditions for tourism are much more suitable than in Poland and Ukraine. However, in summer months (June, July, August), general conditions are unsuitable for tourism, especially its MR and AR forms. In Polish and Ukrainian resorts, annual variations of WSIavg are less manifested than in Serbia. The best, very suitable conditions occur in April and September/October. However, in winter months, weather is at least moderately suitable or suitable for tourism and in summer, suitable (in Poland) and moderately suitable (in Ukraine). These seasonal variabilities of weather allow for reasonable planning of touristic visits depending of individual preferences of tourists.

## Discussion

The results of present research show great seasonal and spatial differences in weather suitability for tourism in compared regions. They are in concordance with the results of previous climate–tourism research carried out in Mediterranean and Central Europe countries. As an illustration, great summer heat stress in Greece and other Balkan countries that cause dramatically unsuitable conditions for tourism was reported by Matzarakis and Mayer (1997), Matzarakis et al. (2014), Zaninović and Matzarakis (2007, 2009), and Zaninović et al. (2006). In the cited research, thermal conditions were assessed using PET and CTIS indicators. Similar finding was also reported by Basarin et al. (2018) for Novi Sad where the number of the days above certain thresholds is increased together with the number of heat waves mostly during the last 15 years. Also, Stojićević et al. (2016) observed a growing trend of annual and seasonal values of PET for 1961–2014 in Banja Koviljaca Spa. Summer decrease in weather suitability for active tourism, both using PET and CTIS indicators, was reported for Odessa (Black Sea coast) by Katerusha and Matzarakis (2015) where period suitable for tourism lasts only from the end of April to mid-May and from the end of September to mid-October (five 10-day periods). Shevchenko et al. (2020) reported that in Kyiv, the period with comfortable bioclimatic conditions is much longer than in Odessa and continues about nine 10-day periods.

The stressful thermal conditions in the summer months in southern Europe were also confirmed when using other biometeorological indices. For instance, analysis of heat load index in Serbia shows that heat stress condition for July 2011 was reported for six different weather stations (Pecelj et al. 2013). Increased heat load values were also observed in 2000 and 2007 in Loznica, Belgrade, Niš, Vranje, Zlatibor and Novi Sad (Мilovаnоvić et al. 2017). Lastly, in the framework of the geo-ecological evaluation of the city of Loznica for the purpose of recreational tourism, the UTCI (2000–2016) shows a marked stress in 2007 in July (Pecelj et al. 2018).

Summer decline in weather suitability for mild and active recreation founded in the present research for Poland and northern Ukraine was previously reported in many papers for Polish tourism resorts (Błażejczyk and Kunert 2011; Mąkosza 2013; Mąkosza and Michalska 2010, 2011; Miszuk 2008; Radzka and Dragańska 2015). Similar summer worsening of MR and AR weather were also detected for other European countries (Błażejczyk and Matzarakis 2008) located mainly in Cfb and Dfb climate zones, e.g. for Austria (Koch et al. 1992; Matzarakis et al. 2007, 2010), Slovenia and Croatia (Zaninović et al. 2006) and for Netherlands (Moreno et al. 2009). All authors link such summer decrease in weather suitability in southern and central Europe with advections of hot tropical and continental air (Gajić-Čapka and Zannović 1997; Koch et al. 1992; Kolendowicz et al. 2018; Nowosad et al. 2013) and with geopotential height (Tomczyk and Bednorz 2019). It makes disturbances in the regulation of heat budget, especially during physical activity (Błażejczyk and Kunert 2011; Epstein and Moran 2006; Fanger 1970; Höppe 1999; Jendritzky 1990).

In general, sun bathing is one of the most expected forms of summer recreation and tourism. However, everyone must remember that in European region, there exists the problem of high values of UVI in summer which can cause health problems, including skin cancer (Błażejczyk and Błażejczyk 2012; Global Solar UV Index 2002). In tourism, resorts should have available information about actual UVI intensity and recommendations on what to do in case of high UVI values.

Our results confirm that tourism is a climate-dependent sector of economy (Andrade et al. 2007, Hamilton 2007, Mansfeld et al. 2007, Scott et al. 2007, Vrtačnik Garbas 2007). Thus, in its future development, the consequences of recently observed climate changes must be taken into consideration (Amelung and Nicholls 2014; Nicholls and Amelung 2008; Endler et al. 2010). The period of climate records used in present research is too short for analysis of changes in trends and to formulate possible projections. However, comparison of data from regions of different climate, especially thermal, regimes make it possible to speculate that if global warming will continue current trends, it can significantly impact weather conditions which influence thermal comfort in tourism resorts. In the studied region, we should expect increase in heat stress days, as is seen in predictions made for Poland (Błażejczyk et al. 2018). Increase in heat stress days is also predicted globally by Pappenberger et al. (2015). This can lead to reduction of weather suitability for active forms of outdoor tourism and this changes must be taken into consideration by tourists and tour operators.

## Conclusions

As outdoor tourism and recreational activities strongly depend on actual meteorological conditions, it is very important to evaluate how weather can influence seasonal and regional streams of tourists and recreationists. According to the results of this study, outdoor recreation should be presented through the active (MR, AR and ST) or passive (SB and AB) forms of recreation.

The present research shows great seasonal variability of weather assessed from the point of view of outdoor recreation. The passive forms of recreation (sun and air bathing) are preferred mostly in months from May till August or September. This is the season usually used for any holiday travels and stays. However, for the active forms of recreation during the summer months, weather is very oppressive, especially in the resorts located in the south of the studied region (Serbia, southern Ukraine). Thus, active forms of recreation are preferred there in autumn, winter and spring months.

Taking into account the weather and climate information that may be relevant for tourists, the results of the research suggest that the southern part of the region is a very good place for passive summer holiday stays. Otherwise, tourists preferring active forms of recreation should consider summer holiday visits in resorts located in northern Ukraine and Poland.

The present research dealt with regional differentiation of climate features for climate. However, in planning of future development of tourism resorts, local authorities should consider also local factors (e.g. green, forests and water areas, shadow, etc.) which can modify the microclimate and help in better adaptation to climate change. This should be the topic of future research.

## Electronic supplementary material

ESM 1 (DOCX 37 kb)
