# Supplementary material for: Weather suitability for outdoor tourism in three European regions in first decades of the twenty-first century
Source: Int J Biometeorol. 2020 Aug 18;65(8):1339–56. doi: 10.1007/s00484-020-01984-z (PMC8346432; doi:10.1007/s00484-020-01984-z)
Supplement: Supplementary file 1 — (DOCX 37 kb) [file 484_2020_1984_MOESM1_ESM.docx]

Supplementary materials

Table S1. An example of weather suitability values of individual weather categories (extracted from BioKlima 2.6 documentation files)

| Weather type | Weather subtype | | | Weather class | | | Individual weather suitanility values | | | | |
| --- | --- | --- | --- | --- | --- | --- | --- | --- | --- | --- | --- |
| Weather indicators | | | | | | |  |  |  |  |  |
| 1 | 2 | 3 | 4 | 5 | 6 | 7 | SB | AB | MR | AR | ST |
| -3 | 1 | C | 0 | 0 | 0 | 0 | 0 | 0 | 0 | 0 | 0 |
| -3 | 3 | T | 2 | 1 | 1 | 1 | 0 | 0 | 0 | 1 | 1 |
| -2 | 1 | C | 0 | 0 | 0 | 0 | 0 | 0 | 1 | 3 | 0 |
| -2 | 1 | C | 0 | 0 | 1 | 1 | 0 | 0 | 0 | 1 | 1 |
| -1 | 1 | C | 0 | 0 | 0 | 0 | 0 | 0 | 1 | 3 | 0 |
| -1 | 1 | C | 0 | 0 | 0 | 1 | 0 | 0 | 1 | 3 | 3 |
| -1 | 1 | C | 0 | 0 | 1 | 0 | 0 | 0 | 1 | 1 | 0 |
| -1 | 1 | C | 0 | 0 | 1 | 1 | 0 | 0 | 1 | 1 | 1 |
| 0 | 1 | C | 0 | 0 | 0 | 0 | 0 | 1 | 3 | 3 | 0 |
| 0 | 1 | C | 0 | 0 | 0 | 1 | 0 | 1 | 3 | 3 | 3 |
| 0 | 1 | C | 0 | 0 | 1 | 1 | 0 | 0 | 1 | 1 | 1 |
| 0 | 2 | C | 0 | 0 | 0 | 1 | 3 | 3 | 3 | 3 | 3 |
| 0 | 2 | C | 0 | 0 | 1 | 0 | 1 | 1 | 1 | 1 | 0 |
| 1 | 1 | C | 0 | 0 | 0 | 0 | 1 | 3 | 3 | 3 | 0 |
| 1 | 1 | C | 0 | 0 | 0 | 1 | 1 | 3 | 3 | 3 | 3 |
| 1 | 2 | T | 2 | 0 | 0 | 0 | 0 | 0 | 0 | 0 | 0 |
| 1 | 3 | C | 0 | 0 | 0 | 1 | 3 | 3 | 3 | 1 | 0 |
| 1 | 3 | C | 0 | 0 | 1 | 0 | 1 | 1 | 1 | 1 | 0 |
| 2 | 1 | C | 0 | 0 | 0 | 1 | 1 | 3 | 3 | 1 | 0 |
| 2 | 1 | C | 0 | 0 | 1 | 0 | 1 | 1 | 1 | 1 | 0 |
| 2 | 2 | C | 0 | 0 | 0 | 0 | 3 | 1 | 1 | 1 | 0 |
| 2 | 3 | C | 0 | 0 | 0 | 0 | 3 | 3 | 1 | 0 | 0 |
| 2 | 3 | C | 2 | 0 | 0 | 0 | 0 | 0 | 0 | 0 | 0 |
| 3 | 1 | C | 0 | 1 | 1 | 1 | 1 | 1 | 0 | 0 | 0 |
| 3 | 2 | C | 0 | 1 | 0 | 0 | 1 | 3 | 0 | 0 | 0 |
| 3 | 2 | C | 1 | 0 | 0 | 0 | 0 | 0 | 0 | 0 | 0 |
| 3 | 1 | 0 | 2 | 1 | 1 | 0 | 0 | 0 | 0 | 0 | 0 |
| 3 | 1 | H | 0 | 0 | 0 | 0 | 1 | 1 | 0 | 0 | 0 |

Table S2. Mean differences in annual frequency of days suitable for particular tourism activities (SB – sun bathing, AB – air bathing, MR – mild physical activity, AR – intensive physical activity, ST – ski tourism) in Poland, 2000-2017

| Pair of stations | SB | AB | MR | AR | ST |
| --- | --- | --- | --- | --- | --- |
| HEL - TOR | 11.2 | 4.6 | 12.2 | 7.9 | -0.3 |
| HEL - WAR | -0.1 | -0.7 | 17.6 | 10.8 | -5.0 |
| HEL - WRO | 27.7 | 14.7 | 11.9 | 8.1 | 3.7 |
| HEL - KRA | 11.0 | 8.3 | 26.6 | 22.9 | -2.3 |
| HEL - JG | 41.4 | 25.3 | 12.5 | -3.1 | -14.2 |
| HEL - HG | 83.9 | 78.9 | 43.8 | -21.3 | -136.7 |
| TOR - WAR | -11.3 | -5.3 | 5.4 | 2.9 | -4.7 |
| TOR - WRO | 16.6 | 10.1 | -0.3 | 0.2 | 3.9 |
| TOR - KRA | -0.2 | 3.8 | 14.4 | 14.9 | -2.0 |
| TOR - JG | 30.3 | 20.8 | 0.3 | -11.1 | -13.9 |
| TOR - HG | 72.8 | 74.3 | 31.6 | -29.2 | -136.4 |
| WAR - WRO | 27.8 | 15.4 | -5.7 | -2.7 | 8.7 |
| WAR - KRA | 11.1 | 9.1 | 9.0 | 12.1 | 2.7 |
| WAR - JG | 41.6 | 26.1 | -5.1 | -13.9 | -9.2 |
| WAR - HG | 84.1 | 79.6 | 26.2 | -32.1 | -131.7 |
| WRO - KRA | -16.7 | -6.3 | 14.7 | 14.8 | -5.9 |
| WRO - JG | 13.7 | 10.7 | 0.6 | -11.2 | -17.9 |
| WRO - HG | 56.2 | 64.2 | 31.9 | -29.4 | -140.3 |
| KRA - JG | 30.4 | 17.0 | -14.1 | -26.0 | -11.9 |
| KRA - HG | 72.9 | 70.6 | 17.2 | -44.2 | -134.4 |
| JG - HG | 42.5 | 53.6 | 31.3 | -18.2 | -122.4 |

Differences statistically significant are marked in yellow

Table S3. Mean differences in annual frequency of days suitable for particular tourism activities (SB – sun bathing, AB – air bathing, MR – mild physical activity, AR – intensive physical activity, ST – ski tourism) in Serbia, 2000-2017

| Pair of stations | SB | AB | MR | AR | ST |
| --- | --- | --- | --- | --- | --- |
| NS – BEL | -1.9 | 16.1 | 11.9 | 7.3 | -4.0 |
| NS – LOZ | 4.0 | 25.7 | 23.3 | 36.6 | -7.4 |
| NS – NIS | 7.4 | 23.3 | 22.6 | 25.8 | -6.6 |
| NS – VRA | -20.5 | -1.5 | -5.3 | 1.9 | -1.4 |
| NS – ZLA | -21.6 | 3.6 | -26.3 | -16.0 | -61.2 |
| BEL – LOZ | 5.9 | 9.6 | 11.4 | 29.3 | -3.4 |
| BEL – NIS | 9.4 | 7.2 | 10.7 | 18.4 | -2.6 |
| BEL – VRA | -18.6 | -17.6 | -17.2 | -5.4 | 2.6 |
| BEL – ZLA | -19.7 | -12.6 | -38.2 | -23.3 | -57.2 |
| LOZ – NIS | 3.4 | -2.4 | -0.7 | -10.8 | 0.8 |
| LOZ – VRA | -24.5 | -27.2 | -28.6 | -34.7 | 6.1 |
| LOZ – ZLA | -25.6 | -22.2 | -49.6 | -52.6 | -53.7 |
| NIS – VRA | -27.9 | -24.8 | -27.9 | -23.8 | 5.2 |
| NIS – ZLA | -29.1 | -19.7 | -48.9 | -41.8 | -54.6 |
| VRA – ZLA | -1.1 | 5.1 | -21.0 | -17.9 | -59.8 |

Differences statistically significant are marked in yellow

Table S4. Mean differences in annual frequency of days suitable for particular tourism activities (SB – sun bathing, AB – air bathing, MR – mild physical activity, AR – intensive physical activity, ST – ski tourism) in Ukraine, 2000-2017

| Pair of stations | SB | AB | MR | AR | ST |
| --- | --- | --- | --- | --- | --- |
| SVI - LVI | -21.6 | -23.9 | -2.0 | -16.8 | -4.7 |
| SVI - KYI | -10.6 | -14.7 | 2.9 | -3.9 | -12.7 |
| SVI - POZ | 8.2 | -3.6 | -0.7 | -31.4 | -90.9 |
| SVI - KHU | -10.3 | -16.0 | 24.9 | 33.4 | -5.8 |
| SVI - UMA | 2.6 | -5.0 | 8.1 | 10.6 | -13.8 |
| SVI - AN | -25.8 | -36.0 | -1.5 | 7.1 | 22.5 |
| SVI - ODE | -18.4 | -26.8 | -7.3 | 11.7 | 23.1 |
| SVI - YAL | -29.3 | -29.5 | 10.8 | 23.3 | 22.8 |
| SVI - MAR | -10.3 | -14.4 | 7.4 | 13.6 | 17.6 |
| LVI - KYI | 11.0 | 9.2 | 4.9 | 12.9 | -7.9 |
| LVI - POZ | 29.8 | 20.3 | 1.3 | -14.6 | -86.2 |
| LVI - KHU | 11.3 | 7.9 | 26.9 | 50.2 | -1.1 |
| LVI - UMA | 24.2 | 18.9 | 10.1 | 27.4 | -9.1 |
| LVI - AN | -4.2 | -12.1 | 0.5 | 23.9 | 27.2 |
| LVI - ODE | 3.2 | -2.9 | -5.3 | 28.5 | 27.8 |
| LVI - YAL | -7.7 | -5.5 | 12.8 | 40.1 | 27.5 |
| LVI - MAR | 11.3 | 9.5 | 9.4 | 30.4 | 22.3 |
| KYI - POZ | 18.8 | 11.1 | -3.6 | -27.5 | -78.2 |
| KYI - KHU | 0.3 | -1.3 | 22.0 | 37.3 | 6.8 |
| KYI - UMA | 13.2 | 9.7 | 5.2 | 14.4 | -1.1 |
| KYI - AN | -15.2 | -21.3 | -4.4 | 11.0 | 35.2 |
| KYI - ODE | -7.8 | -12.1 | -10.2 | 15.6 | 35.7 |
| KYI - YAL | -18.7 | -14.8 | 7.9 | 27.2 | 35.5 |
| KYI - MAR | 0.3 | 0.3 | 4.5 | 17.5 | 30.3 |
| POZ - KHU | -18.5 | -12.4 | 25.6 | 64.8 | 85.1 |
| POZ - UMA | -5.7 | -1.4 | 8.8 | 41.9 | 77.1 |
| POZ - AN | -34.0 | -32.4 | -0.8 | 38.5 | 113.4 |
| POZ - ODE | -26.6 | -23.2 | -6.6 | 43.1 | 113.9 |
| POZ - YAL | -37.5 | -25.9 | 11.5 | 54.7 | 113.7 |
| POZ - MAR | -18.5 | -10.8 | 8.1 | 45.0 | 108.5 |
| KHU - UMA | 12.8 | 11.0 | -16.8 | -22.8 | -7.9 |
| KHU - AN | -15.5 | -20.0 | -26.4 | -26.3 | 28.3 |
| KHU - ODE | -8.1 | -10.8 | -32.2 | -21.7 | 28.9 |
| KHU - YAL | -19.0 | -13.5 | -14.1 | -10.1 | 28.6 |
| KHU - MAR | 0.0 | 1.6 | -17.5 | -19.8 | 23.4 |
| UMA - AN | -28.3 | -31.0 | -9.6 | -3.4 | 36.3 |
| UMA - ODE | -20.9 | -21.8 | -15.4 | 1.1 | 36.8 |
| UMA - YAL | -31.8 | -24.5 | 2.7 | 12.7 | 36.6 |
| UMA - MAR | -12.8 | -9.4 | -0.7 | 3.1 | 31.4 |
| AN - ODE | 7.4 | 9.2 | -5.8 | 4.6 | 0.6 |
| AN - YAL | -3.5 | 6.5 | 12.3 | 16.2 | 0.3 |
| AN - MAR | 15.5 | 21.6 | 8.9 | 6.5 | -4.9 |
| ODE - YAL | -10.9 | -2.7 | 18.1 | 11.6 | -0.2 |
| ODE - MAR | 8.1 | 12.4 | 14.7 | 1.9 | -5.4 |
| YAL - MAR | 19.0 | 15.0 | -3.4 | -9.7 | -5.2 |

Differences statistically significant are marked in yellow
